# Disease monitoring with quantitative serum IgA levels provides a more reliable response assessment in multiple myeloma patients

**DOI:** 10.1038/s41375-021-01180-x

**Published:** 2021-02-23

**Authors:** Alissa Visram, Iuliana Vaxman, Abdullah S. Al Saleh, Harsh Parmar, Angela Dispenzieri, Prashant Kapoor, Martha Q. Lacy, Morie A. Gertz, Francis K. Buadi, Suzanne R. Hayman, David Dingli, Rahma Warsame, Taxiarchis Kourelis, Mustaqeem Siddiqui, Wilson Gonsalves, Eli Muchtar, John A. Lust, Nelson Leung, Robert A. Kyle, David Murray, S. Vincent Rajkumar, Shaji Kumar

**Affiliations:** 1 Division of Hematology, Mayo Clinic, Rochester, MN USA; 2 University of Ottawa, Ottawa Hospital Research Institute, Ontario, Canada; 3 Institute of Hematology, Davidoff Cancer Center, Rabin Medical Center, Petah-Tikvah, Israel; 4 Israel Sackler Faculty of Medicine Tel-Aviv University, Tel-Aviv, Israel; 5 Division of Hematology and HSCT, King Saud bin Abdulaziz University for Health Sciences, Riyadh, Saudi Arabia; 6 Division of Hematology, John Theurer Cancer Center at Hackensack University, Hackensack, NJ USA; 7 Division of Nephrology, Mayo Clinic, Rochester, MN USA; 8 Department of Laboratory Medicine and Pathology, Mayo Clinic, Rochester, MN USA

**Keywords:** Disease-free survival, Myeloma

## Abstract

Unlike IgG monoclonal proteins (MCPs), IgA MCP quantification is unreliable due to beta-migration of IgA MCPs on serum protein electrophoresis (SPEP). The utility of nephelometric quantitative IgA (qIgA) to monitor IgA multiple myeloma (MM) is unclear. We retrospectively studied disease response kinetics using qIgA versus MCPs by SPEP, and developed and validated novel qIgA disease assessment criteria in 491 IgA MM patients. The SPEP MCP nadir occurred a median of 41 (IQR 0–102) days before the qIgA. The median time to achieve a partial response (PR) was shorter using standard IMWG versus qIgA response criteria (32 vs 58 days, *p* < 0.001). Stratification by qIgA criteria, unlike IMWG criteria, led to clear separation of the progression-free survival curves of patients achieving a PR or very good PR. There was a consistent trend toward earlier detection of disease progression using qIgA versus IMWG progression criteria. In conclusion, monitoring IgA MM using MCP-based IMWG criteria may be falsely reassuring, given that MCP levels on SPEP decrease faster than qIgA levels. The qIgA response criteria more accurately stratify patients based on the progression risk and may detect disease progression earlier, which may lead to more consistent measurement of trial endpoints and improved patient outcomes.

## Introduction

Multiple myeloma (MM) is an incurable malignancy arising from plasma cells. Neoplastic plasma cells can secrete monoclonal immunoglobulins which are monitored and used as surrogate markers of disease activity. The serum protein electrophoresis (SPEP) is one of the gold standard laboratory methods for quantifying monoclonal proteins (MCPs). While IgG MCPs most commonly migrate in the gamma fraction of the SPEP, IgA MCPs migrate in the beta fraction of the SPEP in ~30–40% of patients [1, 2]. Beta-migration can lead to an unreliable quantification of the MCP [1, 3, 4] due to the co-migration of physiologic proteins such as transferrin and complement proteins. Therefore, in the 20–30% of patients with an IgA isotype of MM [5–8] the electrophoretic migration of MCP may limit the utility of a SPEP derived MCP in disease assessments. Quantitative nephelometric and turbidometric immunoassays have been studied as a means to monitor IgA MCPs [9] given that monoclonal IgA proteins often predominate over the small normal polyclonal IgA background.

The International Myeloma Working Group (IMWG) response criteria [10], which assess disease response and progression based primarily on changes in the MCP measured by SPEP, are the universally accepted standard for assessing disease response and progression in both the clinical and research setting. Due to limitations in SPEP MCP quantification, the IMWG criteria state that in IgA MM patients the quantitative IgA (qIgA) measurements are preferred for disease assessments. However, this recommendation is based on expert opinion, and the clinical utility of qIgA levels compared with SPEP to assess disease response and prognosticate patient outcomes has not been well studied. Therefore, the aims of this study were to compare the kinetics of disease response in IgA MM patients using both MCP on SPEP and qIgA levels, and to develop and validate response criteria using qIgA.

## Patients and methods

We retrospectively assessed IgA MM patients treated at Mayo Clinic between January 1, 2004 and June 19, 2019. Newly diagnosed MM (NDMM) patients were included in the testing cohort if they were evaluated at Mayo Clinic at MM diagnosis, had serial MCP and qIgA levels available within the first 6 months of diagnosis, and had a qIgA level above the upper limit of normal (ULN) at diagnosis (defined as ≥0.365 g/dL at our laboratory). Patients were included in the relapsed refractory clinical trial validation cohort (RRMM-CT) if they had relapsed IgA MM with serial qIgA levels available from within 6 months of the start of study therapy and had a qIgA above the ULN at study onset. Demographic, baseline laboratory data, disease response and progression data were abstracted manually from the electronic medical record. This study was approved by the Mayo Clinic Institutional Review Board.

### Statistical analysis

Baseline characteristics in NDMM versus RRMM-CT and comparisons between IMWG versus qIgA responses were compared using the Wilcoxon rank-sum test (for continuous outcomes) or chi-square tests (for dichotomous outcomes). Pearson’s correlation test was used to evaluate the association between MCP and qIgA levels. Cohen’s kappa statistic was used to assess the concordance between the classification of disease response by IMWG and qIgA response criteria. The Kaplan–Meier method was used for time-to-event analyses. Progression-free survival (PFS) was defined as the time between the initiation of therapy (first-line therapy for the NDMM cohort and clinical trial therapy for the RRMM-CT cohort) and disease progression (as defined by the IMWG criteria) or death. Overall survival (OS) was defined as the time from progression to death. Uni- and multivariable Cox proportional hazards models were used to assess the risk of progression using IMWG or qIgA criteria, adjusting for risk factors associated with progression. A two-sided *p* value < 0.05 was considered to be significant. All statistical analyses were performed using JMP Pro v14.1 (SAS Institute, Cary, NC).

## Results

A total of 516 newly diagnosed IgA myeloma patients were identified. On review of the medical charts, patients lacking baseline qIgA levels (*n* = 12) or serial qIgA levels measured at our institution within the first 6 months of diagnosis (*n* = 205), or those with qIgA levels within the normal range at diagnosis (*n* = 13), were excluded. Therefore, 286 patients with newly diagnosed MM were included and used as a testing (NDMM) cohort. A second cohort of relapsed or refractory IgA MM patients treated on various clinical trials was used as validation (RRMM-CT) cohort. A total of 223 patients were identified, however, 18 were excluded because they did not have serial qIgA levels available. Therefore, the RRMM-CT cohort consisted of a total of 205 IgA MM patients.

The median duration of follow-up was 46 (IQR 23–73) months in the NDMM cohort. The median duration of follow-up for the RRMM-CT cohort was 31.5 (IQR 12–54) months. In the NDMM and RRMM-CT cohorts, the median MCP on SPEP at treatment initiation was 3.3 (IQR 2.4–4.2) g/dL and 2.3 (IQR 1.3–3.2) g/dL, respectively (*p* < 0.001). The median qIgA at diagnosis was 3.1 (IQR 1.9–4.3) g/dL for NDMM cohort and 2.2 (IQR 1.2–3.3) g/dL at study entry in the RRMM-CT cohort (*p* < 0.001). Nineteen out of the 35 NDMM patients without a measurable serum MCP also had no measurable urine MCP or involved FLC (as defined by the IMWG [10]) at diagnosis, however, the median qIgA at diagnosis amongst these 19 patients was elevated at 0.8 (IQR 0.6–1.1) mg/dL. Further baseline characteristics of both cohorts are outlined in Table 1.

**Table 1:** Baseline characteristics of IgA MM patients included in the testing (NDMM) and validation (RRMM-CT) cohorts.

	NDMM cohort	RRMM-CT cohort
	(*N* = 286)	(*n* = 205)
Age at diagnosis	64 (59–70)	65 (58–71)
Gender		
Male—*n* (%)	179 (63)	120 (59)
Female—*n* (%)	107 (37)	85 (41)
ISS at diagnosis, *n* (%)		
1	64 (23)	46 (24)
2	122 (43)	103 (53)
3	96 (34)	45 (23)
R-ISS at diagnosis, *n* (%)		
1	41 (15)	10 (11)
2	180 (68)	73 (78)
3	44 (17)	11 (12)
FISH, *n* (%)		
Standard risk	164 (70)	61 (63)
High risk	70 (30)	36 (37)
First-line treatment, *n* (%)		
ASCT	148 (52)	77 (104)
IMID + PI + steroid	85 (30)	–
IMID + steroid	75 (26)	–
PI + Alkylator + steroid	75 (26)	–
PI + steroid	21 (7)	–
IMID + Alkylator + steroid	13 (5)	–
Steroid only	9 (3)	–
Alkylator + steroid	6 (2)	–
PI + Anthracycline + steroid	2 (1)	–
Median quantitative qIgA prior to treatment, g/dL (IQR)	3.1 (1.9–4.3)	2.2 (1.2–3.3)
Median MCP prior to treatment, g/dL (IQR)	3.3 (2.4–4.2)	2.3 (1.3–3.2)
Measurable^a^ MCP prior to treatment, *n* (%)	251 (88)	160 (84)
Beta-migrating MCP, *n* (%)	122 (43)	77 (38)

^a^Measurable serum monoclonal protein at diagnosis is defined at ≥1 g/dL.

**Fig. 1:**
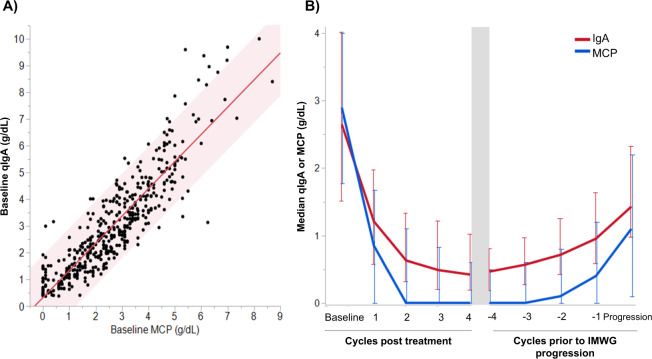
The association between qIgA and MCP measured by SPEP in the combined NDMM and RRMM-CT cohorts. **A** The median change in serial qIgA and MCP levels post treatment and prior to IMWG progression are shown, with error bars indicating the respective interquartile ranges. **B** The correlation of baseline MCP and qIgA levels collected simultaneously for the combined NDMM and RRMM-CT cohorts, the line of best fit and 95% confidence interval are shown in red. The qIgA correlated poorly with MCP when MCP was <1 g/dL (Pearson’s *r* = 0.189, 95% CI −0.033 to −0.394) or >6 g/dL (Pearson’s *r* = 0.4, 95% CI −0.165 to 0.768), however, the correlation improved when MCP was 1–6 g/dL (Pearson’s *r* = 0.857, 95% CI 0.829–0.882).

The baseline MCP levels (measured by SPEP) correlated poorly with baseline qIgA levels when the MCP was <1 g/dL or >6 g/dL, however, the association improved (Pearson’s *r* = 0.857, 95% CI 0.829–0.882) when the MCP was between 1 and 6 g/dL (Fig. 1A). As demonstrated in Fig. 1B, the plateau in MCP occured earlier than the plateau in qIgA, rendering serial measurements of MCP uninformative due to unquantifiable protein levels. Similarly, increases in qIgA above the ULN preceded increases in MCP prior to progression. From this we inferred that changes in qIgA levels may more accurately reflect disease burden. Therefore, we developed response criteria using the change in qIgA levels (as defined in Table 2) in order to assess whether stratifying patients based on changes in qIgA levels could better predict disease outcomes.

**Table 2:** Response criteria based on quantitative IgA.

Assessment	Definition
*Disease response*	
Complete response (CR)	qIgA less than the ULN and sIFE negative
Very good partial response (VGPR)	≥90% decrease in qIgA level and sIFE positive, OR qIgA below the ULN and sIFE positive
Partial response (PR)	50–89% decrease in qIgA level
Minimal response (MR)	25–49% decrease in qIgA level
*Disease progression*	
qIgA_250_ progression	>0.25 g/dL increase in qIgA compared to qIgA nadir, and a ≥25% increase in qIgA level compared to the qIgA nadir, and a positive serum immunofixation
qIgA_500_ progression	>0.5 g/dL increase in qIgA compared to qIgA nadir, and a ≥25% increase in qIgA level compared to the qIgA nadir, and a positive serum immunofixation

*ULN* upper limit of normal (0.356 g/dL), *sIFE* serum immunofixation, *qIgA* quantitative IgA.

### Rate of response in monoclonal protein and quantitative IgA

**Fig. 2:**
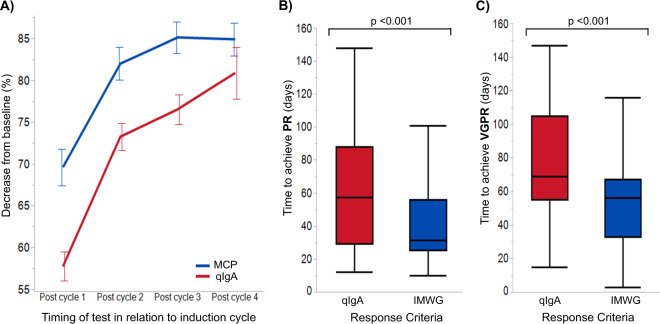
Response kinetics of the NDMM cohort within the first 4 cycles of induction therapy. **A** Line plot showing the mean percent decrease in monoclonal protein (MCP) and quantitative IgA (qIgA) compared to baseline. Patients meeting criteria for progression were excluded. Standard error is indicated by the vertical bars. Box plots comparing the median time to **B** partial response, or **C** very good partial response applying both the qIgA and IMWG response criteria.

The line plot in Fig. 2A is a visual demonstration of the mean percent change in MCP and qIgA from baseline during the first four cycles of therapy in the NDMM cohort. It shows that the MCP plateaus even whilst the qIgA continues to decrease. The median time between initiation of first-line treatment and nadir of MCP was 84 (IQR 43–145) days; the median time to nadir qIgA level was 145 (IQR 90–234) days. The onset of MCP nadir occurred at a median of 41 (IQR 0–102) days earlier than the qIgA nadir. At the onset of MCP nadir, 282 patients had qIgA levels measured; the median qIgA level was 0.3 (IQR 0.2–0.6) g/dL, and 119 (40%) patients had a qIgA level above the ULN (≥0.365 g/dL). In the 240 patients who reached a MCP nadir of 0 g/L, the median qIgA level was 0.2 (IQR 0.1–0.4) g/dL and 79 (33%) had a qIgA level above the ULN. Of the 157 patients with a normal qIgA level, 138 had a concurrent serum immunofixation (sIFE) performed, and 106 (77%) had a detectable IgA MCP on sIFE. Of the 19 patients that had a normal qIgA at MCP nadir but no sIFE tested, 14 (74%) had abnormalities on the SPEP that were suggestive of an unquantifiable residual MCP. Of the 79 patients with a qIgA above the ULN at MCP nadir, all 64 patients with an accompanying sIFE had a detectable IgA MCP, and 13 patients without a concurrent sIFE had abnormalities noted in the gamma (*n* = 4) or beta (*n* = 9) fractions of the SPEP. All patients achieving a complete response (CR) by IMWG criteria (serum and urine immunofixation showing no MCP, as bone marrow biopsies were not performed in all individuals) had a qIgA level within the normal range, with a median qIgA of 0.06 (IQR 0.03–0.1) g/dL.

During the first four cycles of induction therapy, 108 patients achieved a partial response (PR) by both the IMWG response criteria [10] and the proposed qIgA criteria (Table 2). The median time to achieve PR was significantly shorter if response was assessed using the IMWG criteria compared to the qIgA criteria (32 [IQR 25–56] versus 58 [29–88] days, respectively, with *p* < 0.001), as seen in Fig. 2B. Similarly, 95 patients achieved a very good partial response (VGPR) by both IMWG and qIgA criteria within the first four cycles of therapy. The respective median time to achieve a VGPR was significantly shorter using the IMWG criteria compared to the qIgA response criteria (56 [IQR 33–67] versus 69 [IQR 55–109] days, *p* < 0.001), as shown in Fig. 2C. Patients with a beta-migrating MCP had a significantly shorter time to achieve IMWG PR (28 vs 35 days, *p* < 0.001) and IMWG VGPR (44 vs 60 days, *p* = 0.01) compared to patients with a gamma-migrating MCP.

### Defining qIgA response criteria

#### Newly diagnosed multiple myeloma (NDMM) cohort

In order to assess the concordance between the IMWG and qIgA response criteria, the best IMWG response was compared to the best qIgA response (using the nadir qIgA prior to disease progression). The definition of CR was highly concordant between the two staging systems (kappa = 0.89, 95% CI 0.83–0.94), as shown in Supplementary Table 1. Nine patients achieved a best response of VGPR by qIgA criteria, and CR by IMWG criteria. The discordant response classification in these nine patients occurred because the qIgA nadir occurred a median of 42 (IQR 32–91) days before the serum immunofixation was noted to be negative. The discordance between the IMWG and qIgA response definitions was most apparent in the stratification of patients achieving a PR. Of the 134 patients that achieved a VGPR by IMWG criteria, 15 (11%) patients had a discordant response by qIgA criteria, and all 15 were classified as a PR by qIgA criteria.

### Disease response assessments using qIgA and IMWG criteria

#### Newly diagnosed multiple myeloma (NDMM) cohort

**Fig. 3:**
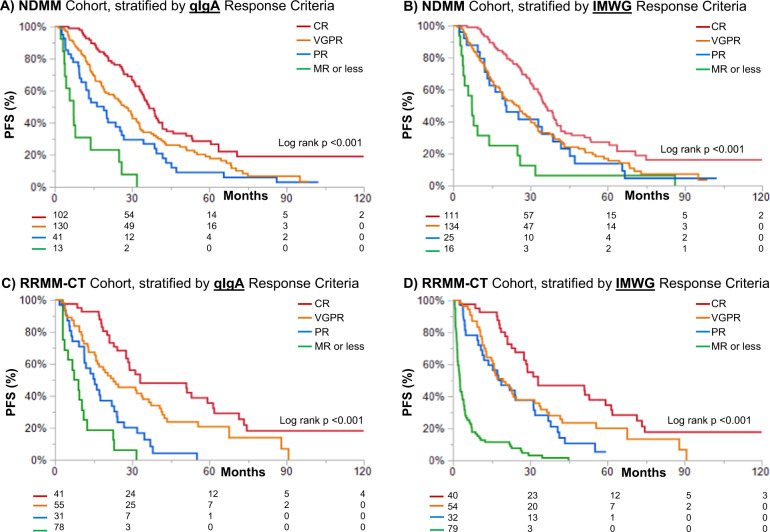
Progression free survival (PFS) stratified by IMWG and qIgA response criteria. The PFS, stratified by the qIgA criteria, is shown for the newly diagnosed multiple myeloma (NDMM) testing cohort (**A**) and relapsed refractory clinical trial (RRMM-CT) validation cohort (**C**). In contrast, the PFS stratified by IMWG response criteria is shown for the testing cohort (**B**) and validation cohort (**D**). CR complete response, VGPR very good partial response, PR partial response, MR minimal response.

The median PFS for the overall training cohort was 29.6 (95% CI 25.5–32.9) months. Stratification of PFS by qIgA response (Fig. 3A) led to a clear separation in the Kaplan–Meier curves for patients achieving a VGPR or PR, as opposed to stratification of PFS by IMWG criteria (Fig. 3B).

In order to understand why patient outcomes were better stratified using the qIgA criteria compared to IMWG criteria, particularly in patients achieving a VGPR or PR by IMWG criteria, we compared the MCP and qIgA levels based on the electrophoretic migration of MCPs in the gamma versus beta region. In patients achieving a VGPR or PR by IMWG criteria, at diagnosis there was no significant difference in the MCP or qIgA levels in patients with MCPs migrating in the beta versus gamma regions. However, at the onset of IMWG best response of VGPR/PR, the median MCP was 0 g/dL regardless of whether the MCP was migrating in the beta or gamma region of the SPEP, but the median qIgA was significantly higher in patients with a beta-migrating MCP compared to a gamma-migrating MCP (0.41 g/dL versus 0.27 g/dL, respectively, *p* = 0.013).

On univariable analysis, attaining a best response of VGPR or better reduced the risk of progression when using either the qIgA (HR 0.46, 95% CI 0.34–0.64, *p* < 0.001) or IMWG (HR 0.55, 95% CI 0.39–0.79, *p* < 0.001) response criteria, when compared to attaining less than a VGPR. However, attaining a CR/VGPR by qIgA criteria was still prognostic of the risk of progression (HR 0.59, 95% CI 0.40–0.86, *p* = 0.006) even after adjusting for age at diagnosis (≥65 verses <65), R-ISS at diagnosis (stage 3 versus stage 1 or 2), measurable MCP at diagnosis (MCP ≥ 1 g/dL versus <1 g/dL), electrophoretic migration of MCP (gamma versus beta region on SPEP), and ASCT status (those that received ASCT versus those that did not). Importantly, there was no significant decrease in risk of progression in patients achieving a VGPR or better by the IMWG criteria after adjusting for the same covariates (HR 0.80, 95% CI 0.52–1.23, *p* = 0.309).

#### Relapsed refractory multiple myeloma patients on clinical trial (RRMM-CT cohort)

Progression free survival, stratified by disease response, was assessed in the RRMM-CT cohort. The median PFS for the whole RRMM-CT cohort was 12.9 (95% CI 10.2–17.6) months. The qIgA response criteria were applied to patients in the clinical trial cohort, and the PFS of this cohort was assessed by stratifying patients according to the qIgA criteria (Fig. 3C) or IMWG criteria (Fig. 3D). This analysis validated the finding that the qIgA response criteria was better able to stratify the PFS outcomes in patients achieving a PR or VGPR when compared to the IMWG criteria.

### Disease progression assessment using qIgA and IMWG criteria

#### Newly diagnosed multiple myeloma (NDMM) cohort

A total of 209 (70%) patients in the NDMM cohort (the training cohort) met criteria of disease progression by IMWG criteria, with a median MCP of 0.7 (IQR 0–1.9) g/dL and qIgA level of 1.0 (0.5–1.8) g/dL at progression. Of these patients, 78 (37%) had no reported MCP on SPEP at the time of progression (21 had new symptomatic bone lesions, 57 met criteria for light chain progression), despite the fact that 41 (53%) of the 78 patients had a qIgA level above the ULN. More importantly, of the 21 patients progressing due to a new symptomatic bone lesion or plasmacytoma in the absence of a detectable MCP, 14 (67%) had a qIgA above the ULN.

In order to assess whether qIgA levels could be used to detect progression earlier than MCP levels, we assessed the time to progression using two different qIgA definitions. We defined qIgA progression as a ≥25% increase in qIgA level compared to the qIgA nadir, and a positive serum immunofixation, and either an absolute increase in qIgA by 0.25 g/dL (the “qIgA_250_” definition) or 0.5 g/dL (the “qIgA_500_” definition), as outlined in Table 2. At qIgA_250_ and qIgA_500_ progression, the median MCP was 0.2 (IQR 0–0.5) g/dL and 0.4 (0–0.8) g/dL, respectively.

**Fig. 4:**
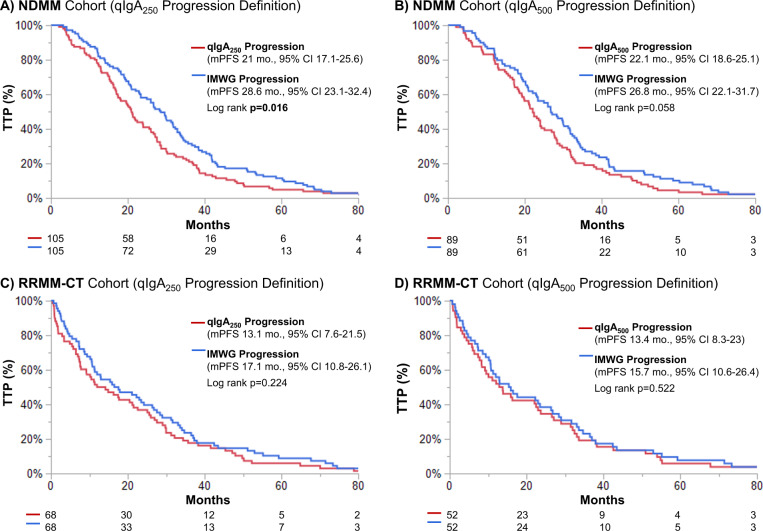
The time to progression (TTP) using the IMWG progression criteria versus the qIgA progression criteria was compared. The TTP was assessed by IMWG progression criteria versus the qIgA_250_ progression criteria (**A**), and qIgA_500_ progression criteria (**B**) in the newly diagnosed multiple myeloma (NDMM) testing cohort. Similarly, the TTP was assessed using the IMWG progression criteria compared to the qIgA_250_ progression criteria (**C**) and qIgA_500_ progression criteria (**D**) in the relapsed refractory clinical trial (RRMM-CT) validation cohort.

We sought to use time-to-event analyses to compare the time to progression with IMWG versus qIgA criteria. Of the 209 patients meeting the IMWG progression criteria, we excluded 63 patients due to a lack of available serial qIgA levels prior to IMWG criteria-based progression, and 41 patients that did not meet criteria for qIgA progression due to a <25% increase in qIgA at IMWG progression. Therefore, in the NDMM cohort, 105 (50%) patients met criteria for both IMWG and qIgA_250_ progression, whereas 89 (43%) patients met criteria for IMWG and qIgA_500_ progression. The median time to progression was significantly shorter using the qIgA_250_ criteria definition compared to the conventional IMWG progression criteria (median time to progression of 21 versus 28.6 months, respectively, *p* = 0.02), as shown in Fig. 4A. Though there was a trend toward earlier assessment of progression with the use of qIgA_500_ criteria compared to IMWG criteria (Fig. 4B), this did not reach statistical significance (median time to progression of 22.1 versus 26.8 months, respectively, *p* = 0.06). The median OS from IMWG progression date was significantly shorter than from qIgA_250_ progression date (33.3 versus 49.8 months, *p* = 0.019), and there was a trend toward shorter median OS from IMWG progression date compared to qIgA_500_ progression date (33.3 versus 52.8 months, *p* = 0.057).

#### IgA multiple myeloma patients on clinical trial

In the RRMM-CT validation cohort, 158 (76%) patients met criteria for IMWG progression, and at progression the median MCP was 1.4 (IQR 0.3–2.4) g/dL and median qIgA was 1.6 (IQR 0.8–2.6) g/dL. Thirty-two (20%) of patients had a MCP of <0.5 g/dL at progression, however, the median qIgA at IMWG progression in these patients was 0.6 (IQR 0.2–0.8) g/dL. Thirteen patients met the IMWG criteria for progression due to new bone lesions or plasmacytoma in the absence of a measurable MCP, and the qIgA was elevated above the ULN in 8 (62%) of these patients.

In order to assess whether the qIgA_250_ and qIgA_500_ progression criteria could predict progression earlier than IMWG criteria, patients meeting both qIgA and IMWG progression criteria were included in the time-to-event analysis. Of the 158 patients meeting criteria for IMWG progression, 68 did not have serial qIgA measurements prior to IMWG progression, and 22 were excluded because they did not meet criteria for qIgA_250_ progression. In the remaining 68 patients who progressed by both the IMWG and qIgA_250_ criteria, there was a trend toward an earlier assessment of progression with qIgA_250_ criteria (Fig. 4C) with a median time to progression of 13.1 months by qIgA_250_ criteria versus 17.1 months by IMWG criteria (*p* = 0.224). Similarly, in the 52 patients progressing by both qIgA_500_ and IMWG criteria, the trend toward earlier progression with qIgA criteria was seen, however, this was not statistically significant (Fig. 4D). There was a trend toward a shorter median OS from the date of IMWG progression compared to the qIgA_250_ progression date (45.9 versus 64.1 months, *p* = 0.080), and from the date of IMWG progression compared to the qIgA_500_ progression date (45.9 versus 63 months, *p* = 0.226).

## Discussion

In this study, we showed that the inaccurate quantification of MCP by SPEP directly affects the clinical response assessment in IgA MM patients. We demonstrate that the artifactual decline in MCP levels overstates response as compared to qIgA levels. Quantitative IgA levels are elevated in 30% of patients even if MCP levels are undetectable, indicating active disease. Furthermore, at the time of IMWG progression ~50% of patients with no detectable MCP on SPEP had qIgA levels above the ULN. In both the NDMM and RRMM-CT cohorts in this study, 60% of patients progressing with new symptomatic bone disease had no detectable MCP yet had qIgA levels above the ULN. These findings highlight the shortcomings of relying solely on SPEP quantification of paraproteins in IgA MM and underscore the importance of monitoring patients with MM using qIgA levels. The serial use of qIgA may also allow earlier detection of disease, which may in turn lead to earlier intervention and an improved prognosis in IgA MM patients. Furthermore, as patients with unmeasurable disease are often excluded from clinical trials, routine incorporation of qIgA levels into clinical trial inclusion criteria will increase the eligibility of IgA MM patients and thereby improve access to novel therapeutic agents. Our study applied the qIgA response criteria only to IgA MM patients with a baseline qIgA above the ULN (≥0.365 g/dL). Therefore, for ease of implementation, we suggest that measurable disease be defined as a qIgA ≥0.5 g/dL (this cutoff would include 98% of our NDMM cohort and 96% of our RRMM-CT cohort).

While the depth of disease response has consistently been shown to prognosticate disease outcomes [11–13], our study showed that stratification of patients by IMWG criteria was not as predictive of the risk of progression when compared to qIgA response criteria, particularly in patients achieving a best response of VGPR or PR. Accurate categorization of disease response is imperative in both clinical trial and routine patient care settings. In clinical trials, the depth of response is commonly used as a study endpoint or as eligibility criteria for randomization to further lines of therapy [14, 15]. Therefore, inaccurate disease response classification may deleteriously affect interpretation of study results. The use of additional lines of therapy to deepen disease response and improve PFS has been demonstrated in the induction and consolidation treatment settings [15–19]. While we do not necessarily advocate altering management to deepen response, it is evident that response classification post treatment influences physicians to provide patients with further lines of therapy for this purpose. In the BMT CTN 0702 trial, 15% of patients had ≥2 lines of therapy pre-transplant, and the median time to registration was 5 (range 2–14) months, suggesting that patients with suboptimal disease response may have received additional therapy [20]. In this context, in IgA MM patients the use of IMWG response criteria may be falsely reassuring to physicians, which may lead to less aggressive management of disease.

Multiple studies have demonstrated that patients with IgA MM have an unfavorable prognosis compared to their non-IgA MM counterparts [21, 22]. However, retrospective studies assessing the gene expression profiles [8], and cytogenetics [23–26] of MM patients have not reported consistent differences between IgA versus non-IgA MM subtypes to explain the difference in prognosis. Given our study’s findings, we propose that the adverse prognosis of IgA MM patients may be in part due to differences in disease management due to misclassification of disease response, or delayed detection of disease due to underestimation of tumor burden.

Previous studies have demonstrated faster response kinetics and deeper IMWG responses in IgA MM compared to non-IgA MM patients [27, 28], however, the biological basis of these observations were not described. We showed that in IgA MM patients with undetectable MCPs at best response, those with MCPs migrating in the beta fraction of the SPEP had significantly higher qIgA levels compared to patients with gamma-migrating MCPs. This illustrates that for beta-migrating MCPs, the proportion of MCP quantified is underestimated due to co-migration with physiologic proteins. The significant heterogeneity in the methodology used to quantitate beta-migrating MCPs between laboratories [3] likely increases the variation in MCP measurements between centers, and further limits the applicability of response criteria that rely on SPEP quantification. Though beta-migrating paraproteins can be more accurately quantified using immunoglobulin heavy/light chain assays [1, 29] or mass spectrometry [30, 31], these methods are not widely available in clinical practice.

Although we did not show statistically significant reductions in the time-to-disease progression using qIgA progression versus IMWG progression criteria, this is likely due to the small number of patients who progressed in our study. There was a consistent trend that using qIgA progression criteria led to earlier detection of progression compared to IMWG progression. Further studies are needed to assess whether implementation of qIgA response and progression criteria leads to improvements in disease outcomes. One could also extrapolate that monitoring qIgA levels in patients with smoldering MM or monoclonal gammopathy of undetermined significance could lead to detection of disease progression earlier, prior to the onset of end-organ damage. Even though the use of qIgA may be more relevant for patients with beta-migrating peaks, it is not easy for community oncologists to make these decisions on a case by case basis, and hence we believe that all qIgA patients could be followed by qIgA. Another important implication of our findings is the use of response criteria for assessment in clinical trials. Depth of response and time-to-event analysis are important endpoints in the efficacy readout of clinical trials and routine use of the IgA for response assessment will allow for consistent assessment of these endpoints.

In conclusion, this study showed that in IgA MM patients the decline in serum MCP levels may be falsely reassuring, as MCP levels decrease faster than qIgA levels. These findings reinforce the IMWG recommendations to use qIgA levels to monitor disease response in IgA MM patients. The proposed and validated qIgA response criteria better stratify patients based on the risk of progression compared to IMWG criteria and may allow for earlier detection of disease progression, which may in turn lead to earlier intervention and improved outcomes for IgA MM patients.

## Supplementary information

Table S1
